# Public Health Risks Associated with Heavy Metal and Microbial Contamination of Drinking Water in Australia

**DOI:** 10.3390/ijerph16203982

**Published:** 2019-10-18

**Authors:** Paul J Molino, Richard Bentham, Michael J Higgins, Jason Hinds, Harriet Whiley

**Affiliations:** 1 Intelligent Polymer Research Institute, Australian Institute for Innovative Materials, University of Wollongong, Wollongong 2500, New South Wales, Australia; mhiggins@uow.edu.au; 2 College of Science and Engineering, Flinders University, GPO Box 2100, Adelaide 5001, South Australia, Australia; Richard.Bentham@flinders.edu.au; 3 Enware Australia Pty Ltd, 11 Endeavour Rd, Caringbah 2229, New South Wales, Australia; Jason.Hinds@enware.com.au

**Keywords:** lead, brass, stainless steel, opportunistic pathogens, *Legionella* spp., non-tuberculous mycobacterium, *Pseudomonas* spp., plumbing material, policy

## Abstract

Recently in Australia concerns have been raised regarding the contamination of municipal drinking water supplies with lead. This is of particular concern to children due to the impact of lead exposure on cognitive development and as such these findings have received much media attention. The response from legislators has been swift, and The Victorian School Building Authority has announced that all new schools and school upgrade works will only use lead-free tapware and piping systems. However, while the immediate replacement of lead-containing brass fittings may seem a logical and obvious response, it does not consider the potential implications on microbial contamination. This is particularly concerning given the increasing public health threat posed by opportunistic premise plumbing pathogens (OPPPs). This commentary explores this public health risk of lead exposure from plumbing materials compared to the potential public health risks from OPPPs. Non-tuberculous mycobacterium was chosen as the example OPPP, and the influence on plumbing material and its public health burden in Australia is explored. This commentary highlights the need for future research into the influence of plumbing material on OPPPs prior to any changes in legislation regarding plumbing material.

## 1. Introduction

The safety and security of Australia’s drinking water supply has been thrust into the limelight over the past several years. This is in no small part due to our dwindling water storage levels, costs associated with engaging mitigating measures such as desalination, and concerns over the contamination of our drinking water by a range of different contaminants. These issues have ignited community interest and concern over the state of our drinking water supply and the potential health impacts. Recently, concerns have been directed to the contamination of our water by lead [1,2,3]. Lead is of particular concern due to its known impacts on the cognitive development of infants and young children, and has been the subject of widespread media attention. In particular the large-scale drinking water lead contamination events in the U.S. in Washington, DC in 2000 [4,5], and Flint, Michigan in 2014 [6] have caused public and political outrage. In Australia, public concerns have been raised over reports of elevated lead levels measured in the recently completed Perth Children’s Hospital in Western Australia [2] and in public drinking fountains in Geelong, Victoria [3]. In both cases, lead leaching from brass tap components has been proposed as the culprit. A recent directive from the Environmental Health Standing Committee (enHEALTH) of the Australian Health Protection Principal Committee (published in July 2018 [7]) outlined approaches to minimize lead exposure from mains drinking water, including the running of taps for 30 secs at first use in the morning to flush fresh water through the tap components. This directive has further heightened community anxiety around the issue of lead in drinking water, and its potential impact on community health. 

Understandably, community response has been swift, and focused on demanding the removal of tap fittings that incorporate lead and replacing them with lead-free alternatives. Legislators have been quick to respond. In January 2019, The Victorian School Building Authority (VSBA) recently announced that all new schools and school upgrade works will only use lead-free tapware and piping systems, for example, only those made from stainless steel, plastic, copper, or lead-free or safe brass. Further to this, in May 2019 the VSBA published a Building Quality Standards Handbook that now specifies that all drinking water fountains must be stainless steel and that “any piping, tapware or fittings that hold or distribute potable water, or form part of a water source where a child could fill a cup or drink bottle for consumption, must be comprised of products that either: do not contain lead, or do not allow contact between brass containing lead” [8]. 

The immediate replacement of lead containing brass fittings may to some present a logical and obvious response. However, this rapid action to legislate has been made with no public debate, and with only limited consultation with scientific experts and industry stakeholders. The failure to consult raises concerns that this action may be hasty, and that it is not based on comprehensive investigation and analysis of the underlying issues relating to lead levels in Australia, including the quantifiable and targeted benefits that this policy change will deliver.

Furthermore, the lack of consultation raises the fear that there have been no considerations of the potential unintended consequences of this policy shift with regards to the potential impacts of replacing brass with other metallic and/or plastic plumbing components at the drinking water outlet. An evidence-based approach should be a basic tenet of formulating mandatory public policy around ubiquitous infrastructure that has the potential to significantly impact public health. This does not appear to be the case in this instance. 

One area that has raised significant concern with several industry stakeholders and those within the scientific community is how replacement materials will impact the contamination of drinking water infrastructure with opportunistic premise plumbing pathogens (OPPPs). Microbial contamination of drinking water infrastructure is a well-recognized emerging public health issue, and concerns regarding its role in the spread of infection has been highlighted in several countries, including the United States [9,10,11], Canada [12], Japan [13,14], Italy [15], and Australia [16] (for review, see [17]). Infections from OPPPs are already considered a public health crisis in the healthcare industry, where waterborne opportunistic pathogens are a major and increasing contributor to hospital acquired infections (HAIs). Hospital tap water has been described “as the most overlooked, important and controllable source of HAI” [7]. The most common OPPPs include *Pseudomonas aeruginosa*, non-tuberculous mycobacterium (NTM), such as mycobacterium avium complex (MAC), *Burkholderia cepacia, Acinetobacter* spp., and *Legionella pneumophilia* [18,19,20,21,22]. 

These pathogens frequently contaminate end-of-the-line plumbing infrastructure, such as taps, valves and shower heads (see Figure S1 for examples of the plumbing fixtures and fittings) and may be influenced by a change in material. Several studies have shown brass to possess antibacterial properties, killing bacteria or impeding bacterial growth and colonization [23,24,25]. The removal and replacement of brass components with other materials may potentially impact the ability for bacteria to adhere and colonize such components. This could lead to increased bacterial contamination of water outlets, and greater risk of infection to the community. 

## 2. Lead Levels in Drinking Water in Australia and Their Origin 

The Australian Drinking Water Guidelines (ADWG) [26] stipulate that lead concentration in drinking water should not exceed 10 µg/L. This value is based on a World Health Organisation assessment that focused on the protection of those most at risk of lead exposure, being young children, infants, and pregnant women. Japan, Ireland, and the European union all have equivalent lead limits for drinking water (10 µg/L). In north America, Canada has a lead threshold of 5 µg/L in drinking water, while it is 15 µg/L in the United states [26]. 

The Washington and Flint lead contamination events in the U.S. were primarily due to changes in water treatment and supply causing an increase in the corrosion of existing and old lead pipes that serviced households from the municipal mains. Unlike the U.S., Australia does not have a widespread issue of water contamination from lead service piping, with lead pipes having been replaced with copper from the 1930s onwards. Rather, lead contamination from brass fittings has been seen as the primary cause for measured elevated lead levels. A 2016 study of heavy metal contamination of drinking water from 212 samples across NSW found lead concentrations to be above the ADWG in 8% of samples, while 5% had copper levels above the permitted value [27]. Surveys of lead in drinking water in other countries have presented similar results, with Germany and Italy recording 6.1%–10.3% and 2% of households measuring lead levels above the 10 µg/L level, respectively [28].

The public health risk of lead contamination from municipal drinking water should be considered in context with other drinking water sources. Several studies from across Australia have identified that rainwater used for drinking purposes frequently contains lead concentrations above the ADWG. One study from South Australia found that 86% of surveyed rainwater tanks contained lead at concentrations above 10 µg/L in at least one sample [29]. Another study conducted in Melbourne found that levels of lead in rainwater tanks exceeded the ADWG by up to 35 times [30]. 

## 3. Contribution of Lead in Drinking Water to Overall Lead Body Burdens

In November 2018, Australia’s chief medical officer, Professor Brendan Murphy, stated in a media release that our drinking water is safe and that there is no evidence of adverse effects on human health from the consumption of lead in drinking water in Australia. He reiterated that the current allowable lead concentration in the guidelines “is very conservative so that it can be sure to protect the most vulnerable people, such as very young children and pregnant women” [31]. Exposures to lead can in fact come via several avenues, including food ingestion, soil and dust ingestion, inhalation, and water ingestion. Over the past 40 years, exposure to lead has fallen dramatically due to several initiatives, the most critical being the removal of lead in petrol and paint, resulting in a huge reduction in community blood lead levels (BLLs) [32]. A 2009–2010 survey by the Victorian Health Monitor revealed that the geometric mean BLL of Victorian adults was 1.45 µg/dL [33], and was comparable to levels measured in similar surveys in the U.S (1.4 µg/dL) [34], France (2.57 µg/dL) [35], and Canada (2.08 µg/dL) [36]. In Australia, as well as in France and Canada, the notifiable BLL is 10 µg/dL. 

Due to the concerns regarding excessive lead exposure to children, a significant amount of work has been undertaken to understand, and model, how lead exposure relates to blood lead levels of individuals, and how best to apportion resources that will most effectively reduce the incidence of high exposure amongst individuals. In Australia, research into child lead levels has been focused on at-risk populations, particularly in the lead mining towns of Mt Isa [37] and Broken Hill, where environmental contamination is thought to be the main driving factor causing elevated BLLs in these communities [38]. A recent small study of 30 children in Mt Isa found that 40% of children had a BLL of ≥5 µg/dL, and 10% had levels of ≥10 µg/dL [39]. In Broken Hill, government intervention in the early 1990s through educational, behavioral, and environmental interventions resulted in a significant increase in the percentage of children with BLLs ≤10 µg/dL, increasing from 13.6% in 1991 to 64.2% in 2001 [40]. 

**Figure 1:**
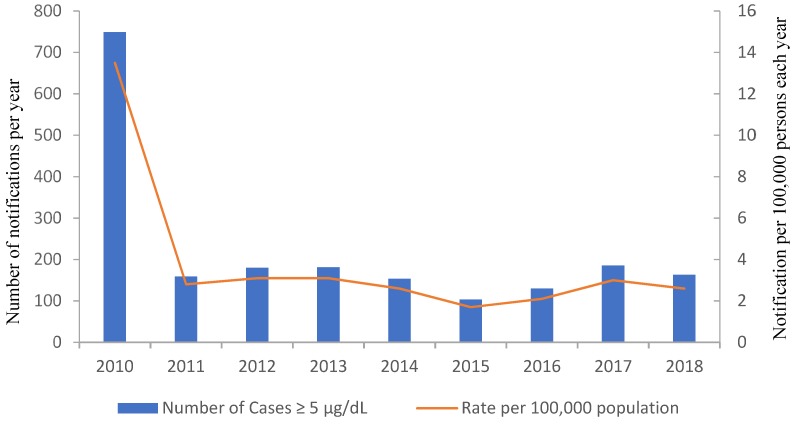
Yearly notifications to Victorian Health of blood lead level measurements of persons reported levels above 5 µg/dL for the years 2010–2018. The number of notifications per 100,000 population are also listed. Source: https://www2.health.vic.gov.au.

**Figure 2:**
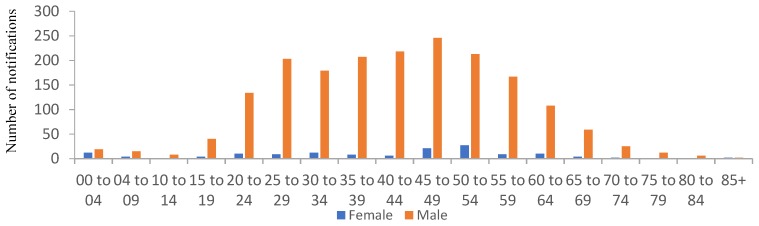
Total number of notifications to Victorian Health of blood lead levels measurements of persons reported above 5 µg/dL for the years spanning 2010–2018, grouped as a function of age group and sex. Source: https://www2.health.vic.gov.au.

Not all states and territories in Australia are required to record BLL events above the level considered elevated (10 µg/dL), and therefore it is difficult to obtain an up-to-date record of BLLs over time at the national level. In Victoria, records show that notifications of BLLs above the moderate value of ≥5 µg/dL have been relatively stable between 2011 and 2018 (Figure 1), with rates of 2.6 per 100,000 population reported in 2018. For the period of 2010 to 2018, the proportion of BLLs above the ≥5 µg/dL level for children less than 14 years is relatively small (Figure 2). The significant difference in the proportion of men presenting elevated BLLs, relative to women, particularly between the ages of 20 and 59 years, has previously been attributed to occupational exposures, which was demonstrated to account for >74% of all notifiable cases between 2011 and 2014. Furthermore, the number of non-occupational exposures that were sourced to lead plumbing was only 6% and 3% of cases in 2011 and 2014, respectively, with no cases found to be caused by lead plumbing in 2012 and 2013 [41]. 

Several studies and reports have highlighted that drinking water does not contribute a significant proportion of the lead body burden, with more than 80% of lead intake estimated to be derived from the ingestion of food, dirt, and dust [42,43]. A recent study based out of the U.S. Environmental Protection Agency [44] has developed a multimedia modelling analysis tool through which to determine the drinking water lead concentrations required to keep children’s BLL (0 ≥ 2 yrs) below specific values, considering lead exposure from water, soil, dust, food, and the air. The authors found that for children between one and two years, soil/dust (77%) and food (16%) ingestion were the greatest contributor to BLLs for children at the upper 90th percentile (≥2.03 µg/mL), with lead in drinking water contributing just 7%. For 0–6 months olds, the use of drinking water in formula preparation increases the contribution of lead to infant BLLs, with soil/dust accounting for 52% of the lead exposure, and water 39% for those at the 90th percentile of the BLL distribution (median 2.66 µg/dL. Their model was used to estimate the drinking water lead concentration required to keep BLL level at or under the 95th percentile considering a target mean BLL of either 3.5 or 5 ug/dL. For 0–6 month olds, keeping BLL levels at or below a mean of 3.5 µg/dL required a lead water concentration at or below 7 µg/L, while this was 17 µg/L for a BLL of 5 µg/dL. For 2–6 year olds, these values are 1 µg/L and 12 µg/L for 3.5 ug/dL and 5 µg/dL BLL values, respectively. This work predicted that for children older than 6 months, lead in drinking water contributes a relatively small proportion of the overall lead body burden, where other exposures, such as from food, dust and soil, are far more problematic. 

## 4. Opportunistic Premise Plumbing Pathogens (OPPPs)

The contamination of drinking water outlets with waterborne pathogens is a growing problem that affects both public and private infrastructure. Water outlets and pipelines can be contaminated by OPPPs present in the source water (such as *L. pneumophila*) or alternatively, at the outlet by individuals washing their hands (such as *P. aeruginosa*) [45]. Once contamination occurs, these pathogens adhere to surfaces and develop or are incorporated into biofilms, which help protect the cells from physical and chemical disinfection [46]. Exposure to these pathogens can occur via ingestion or aspiration of contaminated water during bathing, and drinking, or inhalation of contaminated aerosols from water outlets such as shower heads [42]. This can cause a range of infections in vulnerable people including respiratory, skin and soft tissue, gastrointestinal, blood, and neurological pathologies [17].

Emerging evidence suggests that the number of OPPPs in drinking water is increasing and correlates with the number of individuals vulnerable to infections caused by these pathogens [46]. In the USA, *Legionella* spp. has been identified as the leading cause of drinking water disease outbreaks [47]. With the annual economic cost of infections requiring hospitalizations estimated at $430 million for *Legionella* spp. and $425 million for NTM [48]. One of the biggest challenges facing regulators controlling these pathogens is the difficulties associated with quantifying the associated risks of infection [49]. Without attention to these emerging issues, their public health impact could be underestimated or overlooked compared to comparable or lesser hazards for which the risks can be more easily quantified. 

### Non-Tuberculous Mycobacterium (NTM)

NTM are a particularly pervasive group among the opportunistic bacterial pathogens, often resulting in a slowly progressive and destructive disease which can affect both immune compromised and otherwise healthy individuals [50,51]. Unlike other OPPPs, they can commonly infect children, which makes them an interesting case study to consider in relation to the Victorian Schools situation. In healthy children, NTM can cause cervical lymphadenitis (infection of lymph nodes) which can require surgical excision or antibiotic therapy. Both treatments have complications including reoccurrences of infection [52]. They are also a common cause of pulmonary infection in children with cystic fibrosis [53]. There has been a concerning worldwide increase in the incidence of NTM infections. For example, in Wales and Northern Ireland, NTM cases increased from 5.6 per 100,000 population in 2007 to 7.6 per 100,000 persons in 2012 [54]. In Germany, cases of pulmonary NTM disease increased from 2.3 per 100,000 persons to 3.3 per 100,000 persons from 2009–2014 [55]. In the U.S. the prevalence of NTM disease in the general population was estimated at 1.8 per 100,000 persons in the 1980s [56]. This increased to between 4.1 and 7.2 cases per 100,000 persons [57,58,59] by the early 2000s. 

**Figure 3:**
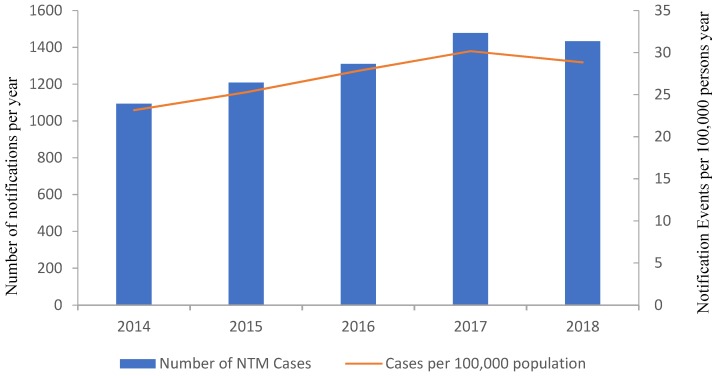
Yearly notifications to Queensland Health of non-tuberculous mycobacterium (NTM) infections for the years 2014 to 2018. The number of notifications per 100,000 population are also listed. Source: https://www.health.qld.gov.au.

**Figure 4:**
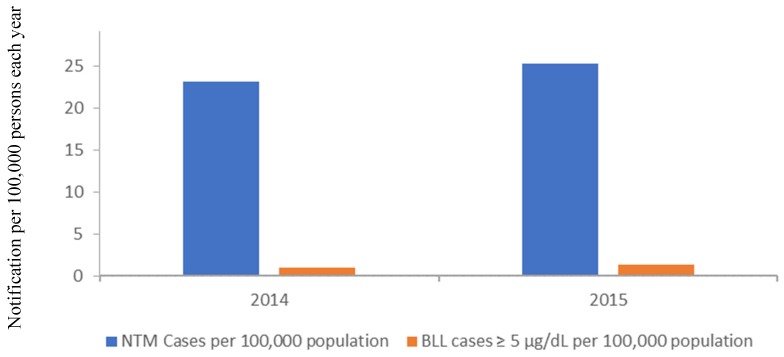
Rate of NTM Infections and blood lead levels (BLL) ≥5 µg/dL in Queensland for the years 2014 and 2015. Source: https://www.health.qld.gov.au.

In Australia, cases of NTM infection are not notifiable in every state or territory, and therefore it is difficult to obtain accurate current records of the rate of NTM infections on a national level. In 2000, a survey of NTM isolated from patients estimated that the national level of NTM infection to be between 5.6 (South Australia) and 71.3 (Northern Territory) cases per 100,000 population, with the national average being 7.5 per 100,000 persons [60]. However, where NTM infections remain a notifiable disease, cases of NTM infection are on the rise, mirroring trends seen internationally. In Queensland, cases have increased steadily in recent years, from 1093 in 2014, 1208 in 2015, 1309 in 2016, 1478 in 2017, and 1433 in 2018 (Figure 3). In 2018, this equated to an infection rate of 28.8 per 100,000 population. In Queensland, publicly available data exists for both the number of reported NTM infections, as well as the number of cases of non-occupational related elevated BLL (≥5 µg/dL) for the years 2014 and 2015 (Figure 4). The rate of NTM infections for those years are significantly greater than the cases of elevated BLL that may require further investigation. 

Sources of NTM may be water, or soil and dust [17], however recent evidence has pointed to plumbing materials and water supplies as being a major contributor to NTM cases. A study in the U.S of 40 households and buildings in 25 different states over a two year period found 78% of water samples taken from drinking water outlets were contaminated with NTM [11]. In Australia, MAC, the most common NTM causing disease, has been found in both chlorine and chloramine disinfected municipal drinking water distribution pipelines [61]. Studies have identified that within the water plumbing system, opportunistic bacterial pathogen contamination is far more prevalent at the end points of the system (i.e. taps, aerators, shower heads) than in the broader pipe plumbing infrastructure [62]), thus providing a reservoir for the bacterial pathogens directly at the user interface. 

While the presence of bacterial contamination of the water outlet does not guarantee users will become infected, it does provide the direct avenue for opportunistic bacteria to infect vulnerable users, such as those with already compromised immune systems through other sickness or disease, the young, and the old. In fact, a direct connection between opportunistic bacterial contamination of drinking water outlets and infections in patients has been established. In Australia, a 2013 study tested the contamination of household water sources for NTM contamination, and compared the genetic profile of the isolated organisms to that isolated from the patient [16]. Therein pathogenic NTM was isolated from 96% of homes (19 of 20 households), where samples were taken from the kitchen, bathroom and shower taps, rainwater tanks, swimming pools, and shower heads. In 35% of cases, the NTM bacteria found in the home was the same as that isolated from the infected patient. A similar study undertaken in the U.S showed that 41% of homes of persons infected with NTM had NTM bacteria present at their drinking water outlets. Furthermore, the NTM isolated from their drinking water plumbing system were identical to that from the infected person [12]. In another study [63], samples taken from showerheads by citizen scientists in the US and Europe were tested for the presence of NTM, with the goal of building a geographic pattern of NTM distribution in different drinking water supplies. Interestingly, hotspot regions were identified in the U.S, where regions presenting a high incidence of NTM contamination of drinking water outlets coincided with regions where NTM infections were most prevalent.

## 5. Plumbing Material

The role of plumbing materials in the proliferation of OPPPs in the drinking water supply has been the subject of increased scientific study of the past several years. A number of studies have shown that copper and copper alloys inhibit the growth and colonization of various waterborne opportunistic pathogens [64,65], often demonstrating significantly reduced numbers of colonizing opportunistic pathogens compared to more benign materials, such as various polymers (polypropylene, synthetic rubbers) and other metals, including galvanized steel and stainless steel. For example, studies comparing bacterial adhesion and biofilm growth on stainless steel, PVC, and copper in a simulated plumbing system over 24 days revealed copper to present the lowest adhered bacterial numbers, followed by PVC, with stainless steel presenting the highest number of bacteria [66]. Another study of *L. pneumophilia* demonstrated that the pathogen was able to adhere to and colonize PVC plastic and copper to a greater extent than brass in a model plumbing system [25].

Currently, studies investigating NTM contamination and the influence of plumbing materials are limited and contradictory [67]. The variation in study findings could be attributed to the number of interconnected variables that influence microbial contamination, including plumbing materials, water chemistry, flow rate, and temperature [68]. Although a recent study surveying the presence of NTM biofilm in shower heads in the U.S and Europe found that material of the shower head significantly impacted on the presence or absence of NTM contamination [63]. This highlights the need for future research to provide a comprehensive understanding of the influence of plumbing material on NTM and other OPPPs. This work needs to be conducted taking into consideration the local conditions, including environmental variables, and should be conducted prior to changing regulation(s). 

### Plumbing Material Quality and Compliance

The Water Mark Certification Scheme is a mandatory certification scheme for plumbing and drainage products to ensure they are fit for purpose and appropriately authorized for use in Australia. For any product to be legally sold in Australia, all manufactures of tapware (faucets) need to attain accreditation to Australian Product Standard AS/NZS 3718 Water Supply—Tapware. All Watermark accredited products are third party certified and obtain a Watermark license to demonstrate its conformity to all product performance and material specifications under the scheme rules. The Australian Building Codes Board manages and administers the Water Mark Certification Scheme nationally. The National Construction Code, Volume Three, requires certain plumbing and drainage products to be certified (listed on the www.abcb.gov.au/product-certification/watermark-certification-scheme Product Database) and authorized for use in a plumbing or drainage installation. 

The Water Mark Certification scheme ensures that plumbing materials comply with a multitude of quality parameters. Within this standard (AS/NZS 3718 Water Supply—Tapware), it is mandatory to meet material requirements as prescribed in AS 2345 Dezincification resistance of copper alloys and AS/NZS 4020 Testing of products for use in contact with drinking water. This evaluates material quality for potential impacts on taste, appearance, growth of aquatic micro-organisms, cytotoxicity, mutagenicity, and extraction of metals. The draft AS/NZS 4020:2019 also includes testing for organic compounds. Any regulatory decisions which counter this Water Mark Certification scheme should be made using a similarly comprehensive and holistic approach to evaluating quality. 

## 6. Conclusions and Recommendations 

Current data suggests that there is limited scientific evidence to support the removal of brass fittings as a means of reducing lead exposures. The complexities of water quality, materials of construction, microbial contamination, and public health risks are not well understood. In view of this intricacy it is ill-advised to focus on one element in these complex interactions and expect a resolution. What is clearly understood is that:

- National and global data does not support that brass fittings are the only source of lead contamination, and should be viewed in respect to the associated human health risk;
- Replacement of existing Watermarked fittings introduces a significant capital cost;
- The potential perceived benefits from the replacement of brass fittings is not balanced against the potential for increased microbial risks from OPPPs;
- The risk of lead exposure is much higher in rainwater compared to municipal water supplies and changes in regulation should reflect this.

Any changes in regulation regarding materials for potable water infrastructure need to be evidence based and must consider all potential risks to public health. Before this can happen there is a need for future research investigating the influence on plumbing material on OPPPs. Given the complexity of this issue and the influence of interconnecting variables on microbial growth, this research needs to be conducted under local Australian conditions. There is also the need for future research to identify the key gaps in knowledge within the plumbing industry regarding OPPPs and plumbing material. This will inform educational campaigns identifying strategies to reduce the risk from OPPPs. 

## Supplementary Materials

The following are available online at https://www.mdpi.com/1660-4601/16/20/3982/s1, Figure S1: Examples of plumbing fixtures and fittings.
