# Supplementary material for: Public Health Risks Associated with Heavy Metal and Microbial Contamination of Drinking Water in Australia
Source: Int J Environ Res Public Health. 2019 Oct 18;16(20):3982. doi: 10.3390/ijerph16203982 (PMC6843933; doi:10.3390/ijerph16203982)
Supplement: Supplementary file 1 [file ijerph-16-03982-s001.pdf]

Supplementary material

Figure S1: Examples of plumbing fixtures and fittings

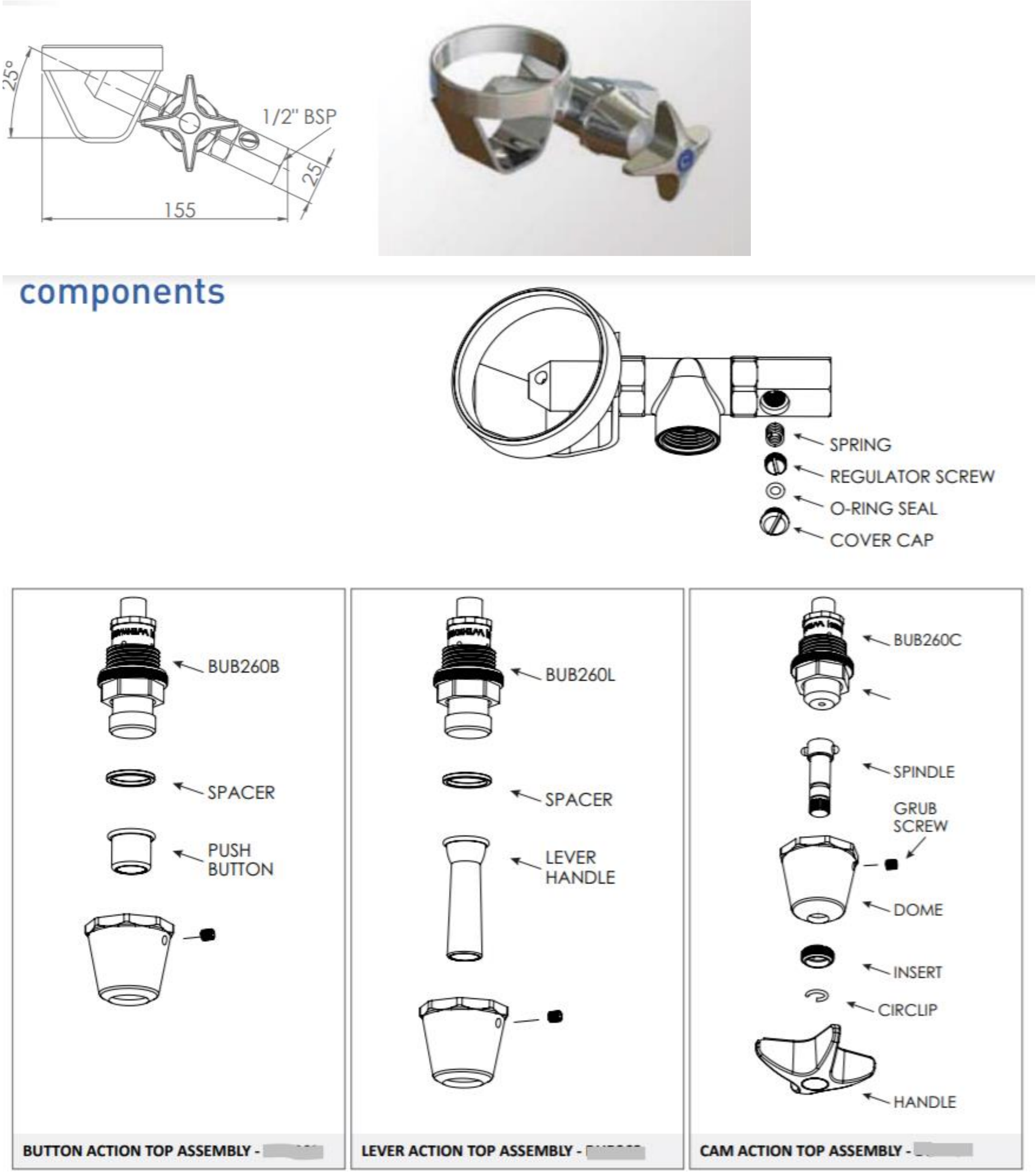

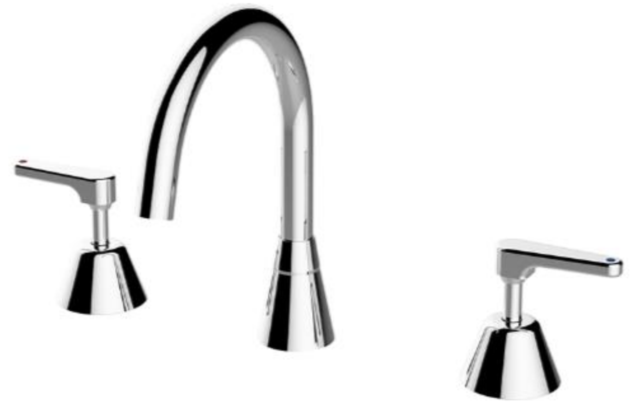

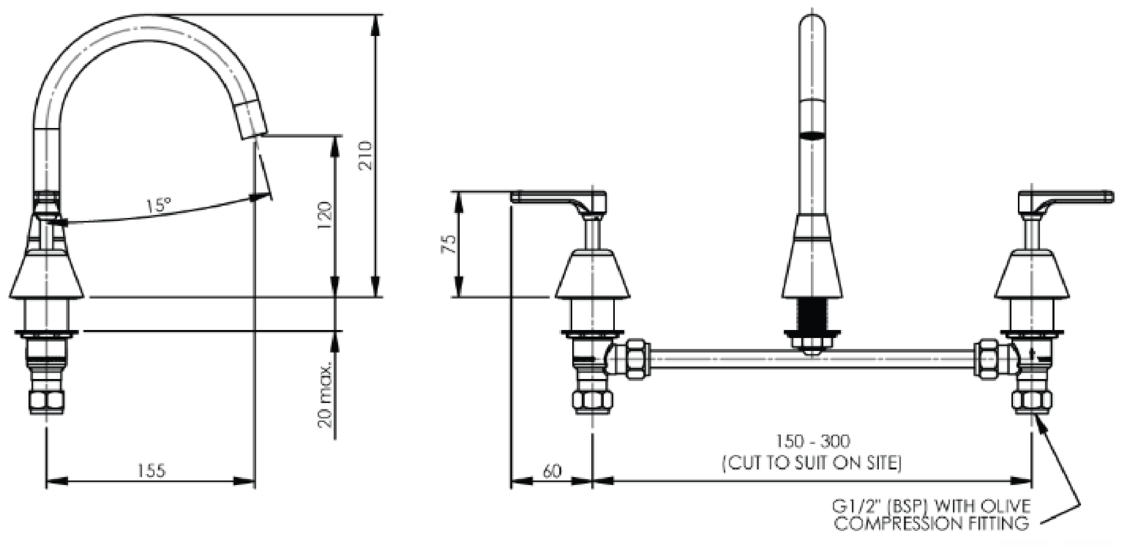
210
120
15°
20 max.
155
75
60
150 - 300
(CUT TO SUIT ON SITE)
G1/2" (BSP) WITH OLIVE
COMPRESSION FITTING

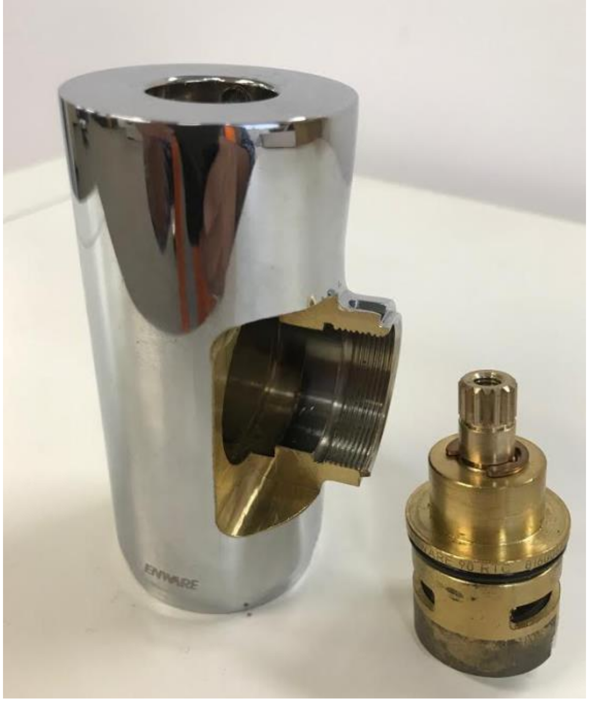
ENWARE
